# A Complementary Filter Design on SE(3) to Identify Micro-Motions during 3D Motion Tracking

**DOI:** 10.3390/s20205864

**Published:** 2020-10-16

**Authors:** Gia-Hoang Phan, Clint Hansen, Paolo Tommasino, Asif Hussain, Domenico Formica, Domenico Campolo

**Affiliations:** 1 Industrial Maintenance Training Center, Ho Chi Minh City University of Technology (HCMUT), 268 Ly Thuong Kiet Street, District 10, C1/268, Ho Chi Minh City 70000, Vietnam; phanhoang@hcmut.edu.vn; 2 The Industrial Traning Center, Vietnam National University Ho Chi Minh City, Linh Trung Ward, Thu Duc District, Ho Chi Minh City 70000, Vietnam; 3 Neurogeriatrics Kiel, Department of Neurology, University Hospital of Kiel, 24105 Kiel, Germany; 4 Laboratory of Neuromotor Physiology, I.R.C.C.S. Fondazione Santa Lucia, 00179 Rome, Italy; PAOLO001@e.ntu.edu.sg; 5 Robotics Research Center, Mechanical and Aerospace Engineering, Nanyang Technological University, Singapore 639798, Singapore; ahussain@articares.com (A.H.); D.CAMPOLO@ntu.edu.sg (D.C.); 6 Centre for Integrated Research, Università Campus Bio-Medico di Roma, 00128 Rome, Italy; d.formica@unicampus.it

**Keywords:** complementary filter, inertia-measurement unit, load cell, motion tracking, sensor fusion, validation, micro-motions, *SO*(3), *SE*(3)

## Abstract

In 3D motion capture, multiple methods have been developed in order to optimize the quality of the captured data. While certain technologies, such as inertial measurement units (IMU), are mostly suitable for 3D orientation estimation at relatively high frequencies, other technologies, such as marker-based motion capture, are more suitable for 3D position estimations at a lower frequency range. In this work, we introduce a complementary filter that complements 3D motion capture data with high-frequency acceleration signals from an IMU. While the local optimization reduces the error of the motion tracking, the additional accelerations can help to detect micro-motions that are useful when dealing with high-frequency human motions or robotic applications. The combination of high-frequency accelerometers improves the accuracy of the data and helps to overcome limitations in motion capture when micro-motions are not traceable with 3D motion tracking system. In our experimental evaluation, we demonstrate the improvements of the motion capture results during translational, rotational, and combined movements.

## 1. Introduction

Optical motion capture technology is a valuable tool when quantifying human movements and clinical, biomechanical, and industrial applications require high system accuracy [1]. While motion capture is often tied to high costs, over the last years less expensive systems have been developed to measure human or robotic motion including goniometers [2], accelerometers [3,4,5], inertia-based and electromagnetic sensors [6,7,8]. A variety of research has been conducted to report characteristics of selected sensor systems and/or to validate new technical devices. In industrial applications such as tooling, specific motion paths are important and unforeseen or unpredictable perturbations influence the final results [9]. Quantifying small motions is a challenging task especially in tooling tasks where vibrations or micro-motions regularly occur and affect the movement itself. In order to measure those vibrations, 3D motion capture can be used by applying local and global optimization techniques [10,11], but the system itself is associated with high costs and lower acquisition frequency compared to, e.g., accelerometers. Therefore, the question arises how to properly measure micro-motions that are characterized by small amplitudes and high frequencies.

Microelectromechanical systems, highly miniaturized and low-cost inertial measurement units (IMUs) can be used to measure accelerations, in particular gravity, and the earth-magnetic field. This information can be merged to reliably estimate orientation in 3D space [12]. In this process, the body-fixed angular velocity derived from gyroscopes (also typically embedded in IMUs) is key to separate otherwise indistinguishable earth acceleration (9.81 m/s${}^{2}$ and constant only in a earth-fixed frame) from accelerations due to actual motion of the IMU [13,14,15]. In other words, 3D orientation is estimated by filtering out accelerations due to the IMU’s actual motion. This information, however, should not be discarded since after representing accelerations in space-fixed coordinates, a time integration could be performed once to obtain IMU velocities or even twice to estimate spatial displacements. This method, however, is hardly applicable in practice since noise and errors will result in unbearable drift effects due to the time-integration process. In this work, we show how this information can still be used in combination with marker-based motion capture technologies.

The combination of both technologies is especially important when capturing complex human movement [16]. With the development of depth cameras, various research has been performed to combine information from camera based systems with IMUs (see, e.g., in [17]). The integration of IMUs into the camera feed increases the accuracy of the human tracking [18] and point into a general problem of camera based motion capture. 3D markers may be occluded as people move or the number of cameras is simply not sufficient enough to capture each marker throughout the whole experiment. The integration of IMUs could therefore be a very interesting choice as the orientation of the limbs can be accurately tracked and combined with the position data from the motion capture creating a very robust solution. Labeling and tracking missing markers is very time-consuming and often the bottle neck in such research cases.

Marker-based motion capture technologies are primarily designed to provide space-fixed coordinates of a series of markers typically fixed onto a moving object. While marker positions can, in principle, be obtained with high accuracy, detecting the orientation of an object requires at least three markers and the orientation errors also depend on the distance between markers and their initial placement. Furthermore, the inherently low frequency of camera-based systems makes it not ideal to detect fast motions. In this sense, IMUs and marker-based motion capture systems complement one another as the former primarily detects orientations and capable of performs better at higher-frequencies (positional drifting being the main issue at low frequencies), while the latter primarily detects positions and performs better at lower frequencies.

Both camera-based and IMU technology have improved over the last decade and the acquisition frequency of cameras can exceed 500 frames per second, being high enough for human movement analysis. However, the implementation of IMUs could in this case also help to detect specific time points during the movement analysis such as heel strikes during running. The detection of such events is of higher accuracy compared to the optical estimation and hence the integration of IMUs allows to detect such events also in the aforementioned case of marker loss. Another application with much higher frequencies are industrial tasks such as tooling. The interaction of the tool with a work piece produces high frequencies that can if exposed to it for too long to vibration white finger [19]. In order to prevent such syndromes the exposure time is limited and regulated. When measuring such tasks the vibration frequency can exceed 2000 Hz, which is difficult if not impossible to detect with a camera-based system.

We previously presented a method to filter motion capture signals and to eliminate the gravitational biasing induced by the mass of an object [20]. In this paper, we propose an experimental set-up capable of capturing micro- and macro-motions. In addition, we compare our results with an external load cell, serving as the ground truth.

## 2. Multimodal Sensor Fusion for Motion Tracking

**Figure 1:**
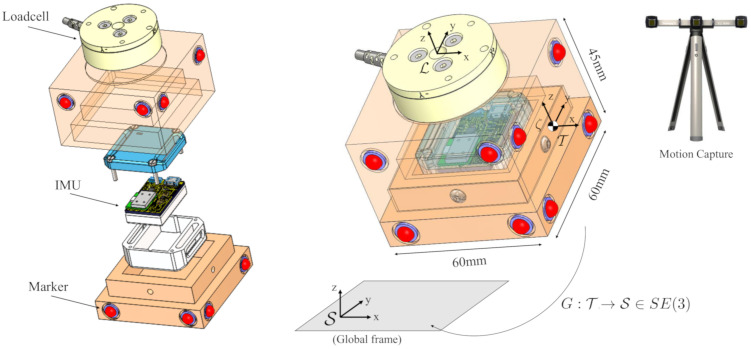
Conceptual design of the instrumented box: a loadcell attached on the top of the box; the inertial measurement unit (IMU) is located inside, at the center while the infrared markers allow capturing 3D position and orientation of the box. Attaching markers on the top and side of the tool. Assuming that the marker’s position on the tool is known from the computer-aided design (CAD) drawing.

In order to capture micro- and macro-motions of an object, we propose a complementary filter algorithm. To validate the assumptions and compare the measured motions with a ground truth, we designed a 3D printed object that includes motion capture markers, an IMU, and a loadcell (see Figure 1). As explained below, the loadcell will be used to sense apparent forces derived from non-inertial accelerations, serving as ground truth for comparison with purely kinematic information derived from IMU and Motion Tracker.

A marker-based motion capture system (VZ4000, Phoenix Technologies Inc., Vancouver, BC, Canada) is used to sense the 3D location of eight infrared markers rigidly attached to the object. To track the micro-motion the IMU (will be presented in Section 3.1, 3-axis gyroscope & 3-axis accelerometer) is located inside the object to measure the change in rotation and the accelerations. A loadcell (ATI mini 40, range $F_{X,Y}$: ±80 N, $F_{Z}$: ±240 N, $T_{X,YZ}$: ±4 Nm) is placed on top of the object to capture the dynamic vibration (i.e., forces) during the movement. The loadcell serves as the ground truth to compare the estimated acceleration based on the complementary filter algorithm. As shown in Figure 1, four major reference frames shall be considered:

- task frame T: located at the center of one of the side face of the box.
- loadcell frame L: attached to the center of the mounting-plate of the loadcell.
- IMU frame: located at the center of the box.
- global frame S: defined by the motion tracker, via external markers mounted on the workbench.

The x, y, and z-axis of task frame, loadcell frame, and IMU frame are simply remains aligned together as shown in Figure 1.

Marker-based optical motion capture is a widely used technology to track 3D positions and orientations. Marker occlusions are a commonly known problem as each marker must be visible to at least two cameras in each frame to establish its position. Dorfmuller, in [21], used an extended Kalman filter (EKF) to predict the missing markers based on previous marker information, while Welch et al. in [22], used the EKF to resolve occlusions based on the skeletal model. These methods require manual intervention or become ineffective in cases where markers are missing for an extended period of time. In this section, we will propose an algorithm capable of overcoming these shortcomings.

### 2.1. Damped Least Square Algorithm for the Filtering Motion Tracker’s Signal

In kinematics, the set of all possible three-dimensional rotations and translations of a rigid body can be described via 4×4 homogeneous matrices constituting the Special Euclidean matrix Lie group, denoted by SE(3) for 3-dimensional Euclidean spaces  [23,24]. In particular, the *pose* of a rigid object in task space T can be described in reference frame S via a homogeneous transformation matrix *G*:

(1) $$SE\left(3\right)=\left\{G\left|G=\left[\begin{array}{cc} R & p\\ 0 & 1 \end{array}\right]\right.,R\in SO\left(3\right)\right\}$$

where $p\in R^{3}$ and $R\in R^{3\times 3}$, respectively, are the translation vector and the rotation matrix representing the axes of the object frame T in global coordinates S. The matrix Lie group SO(3) of Special Orthogonal matrices, is the group of all rotations about the origin of three-dimensional Euclidean space $R^{3}$ under the operation of composition:

(2) $$SO\left(3\right)=\left\{R\in R^{3\times 3}|R^{T}R=I,det\left(R\right)=1\right\}$$

The associated Lie algebra so(3), i.e., the tangent space to the group SO(3) at the identity matrix, is the set of antisymmetric matrices and is defined as

(3) $$so\left(3\right)=\left\{\Omega \in R^{3\times 3}|\Omega =-\Omega^{T}\right\}$$

A *hat* operator $\hat{\cdot }:R^{3}\to so\left(3\right)$ maps 3D vectors (such as body angular velocities) into skew-symmetric matrices. In particular, every 3D vector $\omega ={\left[\omega_{1}\;\omega_{2}\;\omega_{3}\right]}^{T}$ is into one-to-one correspondence with a skew-symmetric matrix:

(4) $$\omega =\left[\begin{array}{c} \omega_{1}\\ \omega_{2}\\ \omega_{3} \end{array}\right]⟼\hat{\omega }=\left[\begin{array}{ccc} 0 & -\omega_{3} & \omega_{2}\\ \omega_{3} & 0 & -\omega_{1}\\ -\omega_{2} & \omega_{1} & 0 \end{array}\right]$$

The inverse of the “hat” operator is referred to as “vee” operator and denoted by ${\left(\cdot \right)}^{\vee }$. The skew symmetric $\hat{\omega }\in so\left(3\right)$ is the matrix logarithm of rotation matrix *R*: $\hat{\omega }=log\left(R\right)$ and $R=exp(\hat{\omega })$. Occasionally, we shall use $log_{\vee }$ as shorthand for ${\left(log\left(\cdot \right)\right)}^{\vee }$, i.e., $\omega =log_{\vee }\left(R\right)$.

In this section, eight infrared markers are located on a rigid object with the relative position known based on computer-aided design (CAD) drawing (see Figure 1). The position of *i*th marker in the task space T is constant and can be defined as

(5) $$m_{i}^{T}={\left[\begin{array}{ccc} m_{ix} & m_{iy} & m_{iz} \end{array}\right]}^{T}$$

The position of the *i*th marker with respect to the global frame S changes with *G* and can be determined as

(6) $$\left[\begin{array}{c} m_{i}^{S}\\ 1 \end{array}\right]=G\left[\begin{array}{c} m_{0i}^{T}\\ 1 \end{array}\right]$$

**Figure 2:**
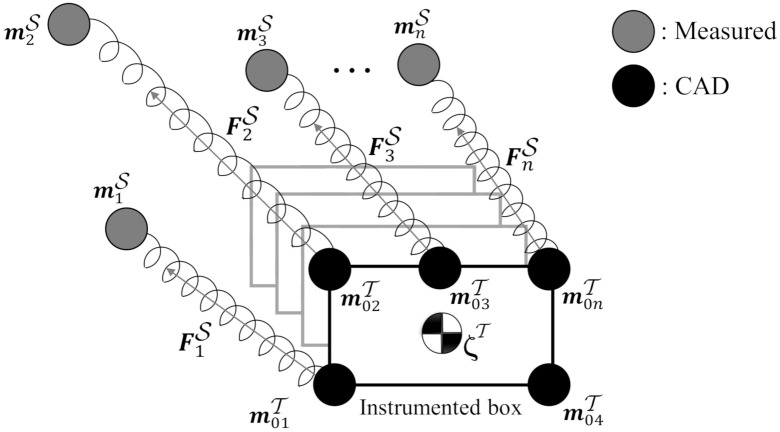
Spring-Markers system: In a time interval, the different between the position estimated and position from CAD of each marker cause the force pull the tool back to the right position.

One way to solve the marker occlusion problem, is the *damped least square* (Figure 2) method which considers each marker as a spring with stiffness $k_{i}$. When the position of the marker changes, there is a difference between its previous position $m_{i}\left(t-1\right)$ and its current position $m_{i}\left(t\right)$, and consequently $k_{i}$. The time interval between two samples $t_{n}\leq t\leq t_{n+1}$ is divided into *N* intervals with the same length. If the marker deviates from its correct position, due to the stiffness a force will pull each marker back to the correct position. The larger the interval *N*, the higher the accuracy of the algorithm.

Let $G^{*}$ be the estimate pose of the tool frame T, then from Equation (6), the estimated position of the *i*th marker is given as

(7) $$\left[\begin{array}{c} m_{i}^{*S}\\ 1 \end{array}\right]=G^{*}\left[\begin{array}{c} m_{0i}^{T}\\ 1 \end{array}\right]$$

we can define this as

(8) $$\left\{\begin{array}{c} F_{i}^{S}=k_{i}\left(m_{i}^{S}-m_{i}^{*S}\right)\\ M_{i}^{T}=\left(R^{-1}F_{i}^{S}\right)\times \left(\zeta^{T}-m_{0i}^{T}\right) \end{array}\right.$$

where $F_{i}^{S}$ is the force in the reference space, $M_{i}^{T}$ is the torque in task space of marker *i*th, and $\zeta^{T}\in R^{3}$ is the center of mass in task space T measured from the mechanical design.

The angular velocity of the tool generated by this change can be defined as [23]

(9) $$\omega^{T}=b_{r}^{-1}\sum M_{i}^{T}$$

where $\omega^{T}\in R^{3}$ is the tools angular velocity vector. The damping of rotation $b_{r}$ will be identified later. To avoid inconsistencies due to numerical integration, geometric integration is preferred, following the work in [23]:

(10) $$R_{n+1}=R_{n}exp\left(\omega^{T}\Delta T\right)$$

The exponential $exp(\omega^{T}\Delta T)$ can be computed via the Rodrigues’ formula [25], which does not drift from SO(3):

(11) $$R_{n+1}=R_{n}\left(I+\alpha_{n}\frac{\Delta T}{N}{\hat{\omega }}_{n}^{T}+\beta_{n}\frac{\Delta T}{N}{\left({\hat{\omega }}_{n}^{T}\right)}^{2}\right)$$

where

(12) $$\left\{\begin{array}{c} \begin{array}{c} \alpha_{n}=\frac{\sin\left(\|\Delta T{\hat{\omega }}_{n}^{T}\|\right)}{\|\Delta T{\hat{\omega }}_{n}^{T}\|} \end{array}\\ \beta_{n}=\frac{1-\cos\left(\|\Delta T{\hat{\omega }}_{n}^{T}\|\right)}{\|\Delta T{\hat{\omega }}_{n}^{T}{\|}^{2}} \end{array}\right.$$

Similar to the rotation, a change in translation of the origin of T in the reference frame S for each time interval:

(13) $$p_{i}^{*}=\frac{\Delta T}{N}\frac{1}{b_{p}}\sum F_{i}^{S}$$

where $b_{p}$ is damping of linear translation and *N* is the number of intervals between two samples of data.

The translation between current and previous position can be defined as

(14) $$p_{n+1}=p_{n}+p_{i}^{*}=p_{n}^{S}+\frac{\Delta T}{N}\frac{1}{b_{p}}\sum F_{i}^{S}$$

For convergence with a time constant τ, set

(15) $$\begin{array}{c} b_{p}=\tau \cdot k\\ b_{r}=\tau \cdot k\cdot L^{2} \end{array}$$

where *L* is a characteristic length of the object, can be defined as

(16) $$L=\frac{\sum_{i}∥\zeta^{T}-m_{0i}^{T}∥}{N_{mrk}}$$

where $\zeta^{T}$ is the center of mass of the object and $N_{mrk}$ is the number of markers.

For implementation purposes the psuedocode is presented in Algorithm 1:

**Algorithm 1:**Damped least square algorithm for the filtering motion tracker’s signal.

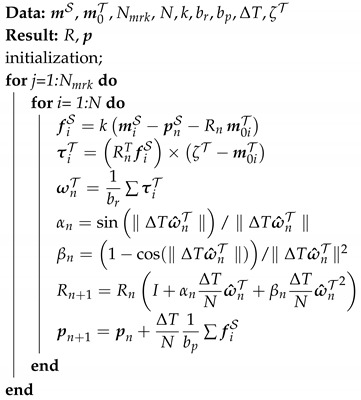



### 2.2. Experimental Verification of the Filtered Motion Capture Signal

**Figure 3:**
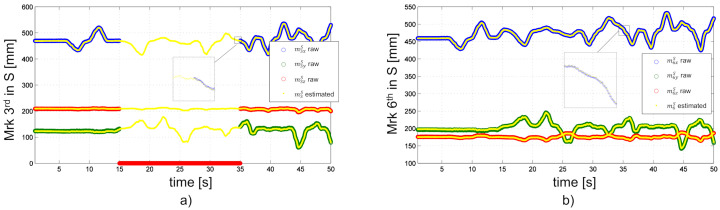
(**a**) Marker 3rd is invisible in 20 s and (**b**) Marker 6th is visible. Estimated marker position based on the complementary filter in x, y and z-direction.

Measuring Object’s position: To test and validate the filter, eight optical markers are attached to a rigid object (see Figure 1). Four additional markers from the origin of the global coordinate frame S. The position of the markers is defined via the computer-aided design (CAD) drawing. The object is moved randomly involving translations and rotations with the motion capture system tracking at 50 Hz. All markers are visible during this experiment. To verify the filter algorithm, the position data of four markers is manually manipulated i.e., the markers 3rd can be considered invisible to the camera system for a continuous time of 20s. Figure 3 shows that the complementary filter can reconstruct the missing markers. The experimental test to verify the damped least square filter algorithm is presented in [20].

## 3. Complementary Filter on SE(3)

In the previous section, a model is developed to filter the motion capture data. The motion capture can detect large rotations and translations at low frequencies, but micro-motions may stay undetected when the amplitudes are too small and the frequencies too high. This section proposes an algorithm capable to capture both the micro- and macro-motions on SE(3).

Complementary filters traditionally apply when redundant measurements are available [26] and combining all available information in order to minimize the instrumentation error. They are also used to fuse information from multiple sensors and correct the output based on the sensed inputs. Although the Kalman filters can be extended (EKF) to nonlinear cases, they fail in capturing the nonlinear structure (e.g., rotations of a rigid body) which can be detected using complementary filters [13,14,15,27]. Therefore, the question arises how to properly measure micro-motions that are characterized by small amplitudes and high frequencies. IMUs can be used to measure orientations, accelerations, and the magnetic field.

Both the IMUs and motion capture can detect the orientation of an object in space. While motion capture system have high 3D resolution at reasonably high acquisition frequency they are outperformed by IMU in terms of temporal and technical resolution. Accelerometers and gyroscopes may have high temporal resolution but are known to show signal drift over time. A combination of both systems could improve the acquisition of slow major movements (3D motion capture) and micro-motions (IMUs) at the same time.

### 3.1. High-Frequency Motion Capture Imu Sensor Specifications

**Figure 4:**
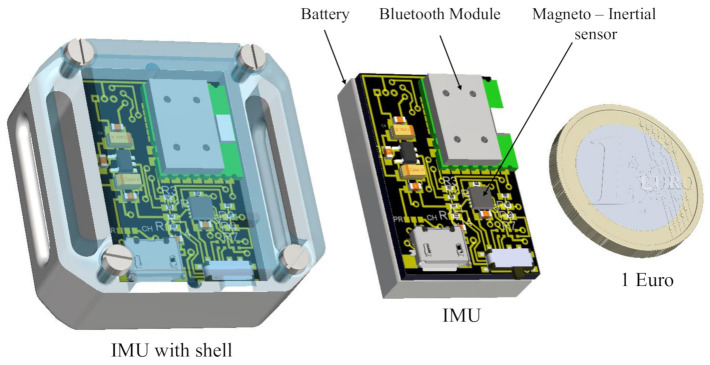
The IMU sensor unit.

In this paper, a customized IMU described in the following is used together with motion capture to improve the acquisition of slow major movements (3D motion capture) and micro-motions simultaneously. The IMU sensor unit is mainly composed by (i) a 9-axis magneto-inertial sensor, the LSM9DS0 manufactured by STmicroelectronics, Inc., which embeds 3 axial accelerometers, gyroscopes and magnetometers. The LSM9DS0 is designed with a small outline (a 4 × 4 mm LGA24 package) and provides digital access to sensor data via I2C serial communication; (ii) a 16-bit microcontroller, the dsPIC33FJ64GP202 by Microchip, Inc., which collects the data from the sensor, and sends it to the Bluetooth module for wireless transmission. The microcontroller also processes the data with a quaternion-based complementary filter [28] in order to estimate the 3D orientation of the sensor, and transmits both raw data and computed quaternions with a sampling frequency of 200 Hz; (iii) a Bluetooth module, the SPBT2632C2A, by STmicroelectronics, Inc., guarantees the wireless communication from the sensor unit to a remote laptop via a Virtual COM Port (VCP); (iv) a rechargeable Li-Po battery with a capacity of 260 mAh, which assures the power supply with at least 4 h of continuous data streaming. The overall dimensions of the IMU sensor unit are 35 × 35 × 22 mm (see Figure 4).

### 3.2. Complementary Filter

Based on the complementary filter on SO(3) for dynamic attitude estimation presented by Campolo et al. [23], the measurement inputs are the rigid body orientation obtained from the motion capture system and the angular velocity measured by the IMU to estimate the kinematics of the rigid object on SE(3). An experimental validation is proposed to verify the filter algorithm will be presented in the next section. The following identities are used.

- The rotation $R_{MT}$ and $R_{IMU}$ denotes the relative orientation of the instrumented box T with respect to the global frame S which is measured by using, respectively, the motion tracker and IMU.
- Vectors $p_{MT}$ presents the translation of T frame in global frame S.
- $\omega_{gyr}$ denotes the angular velocity of the rigid object measured by the IMU.
- $a^{T}$ denotes the output of the accelerometers embedded in the IMU and are expressed in moving frame coordinates.
- $f_{v}^{L}$ denotes the oscillation force which is caused by the plate of the loadcell.

**Figure 5:**
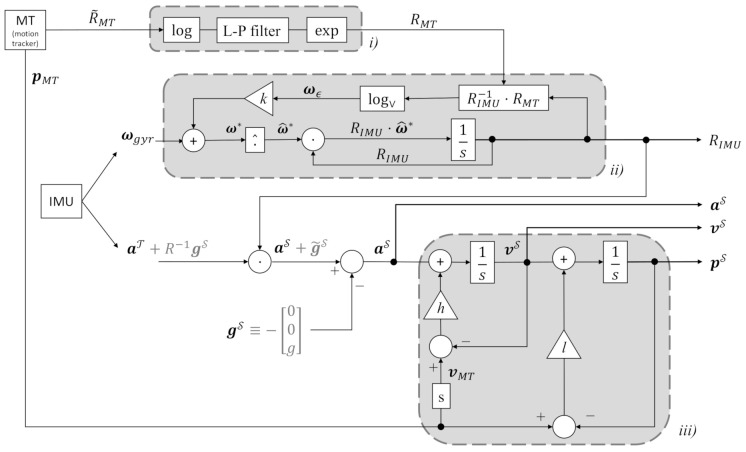
SE(3) complementary filter used to estimate 3D orientation $R_{IMU}$ as well as space-frame positions $p^{S}$, velocities $v^{S}$, and accelerations $a^{S}$ from motion tracker (MT) and IMU raw data. The three gray blocks represent (i) a low-pass filter on SO(3), (ii) a nonlinear SO(3) complementary filter combining low-pass filtered 3D orientations $R_{MT}$ from the motion tracker with angular velocities $\omega_{gyr}$ from the gyroscopes, and (iii) a cascade of two linear complementary filters to estimate space-fixed velocities $v^{S}$ and positions $p^{S}$ from space-fixed accelerations. The operations outside of the gray boxes simply represent body-frame accelerations $a^{T}$ into space-frame accelerations $a^{S}$ from the constant the gravitational bias $g^{S}$ can be simply subtracted.

Given a rigid object with a defined object frame T, the rotation $R_{MT}^{S}$ measured by using the motion tracker presents the relative orientation of the rigid object frame T with respect to the global frame S. The gray block (i) in Figure 5 shows how raw data from motion capture ${\tilde{R}}_{MT}$ are geometrically preprocessed to generate a filtered output $R_{MT}$ to be fused with IMU angular velocity $\omega_{gyr}$. Such a sensor fusion takes place in the SO(3) complementary filter shown in the block (ii) in Figure 5. Specifically, the rotation $R_{IMU}$ represents the estimated orientation of the rigid object and is defined by the following time-continuous equations,

(17) $$\left\{\begin{array}{l} {\hat{\omega }}_{e}=log\left(R_{IMU}^{-1}R_{MT}\right)\\ \omega^{*}=\omega_{gyr}+k\omega_{e}\\ {\dot{R}}_{IMU}=R_{IMU}{\hat{\omega }}^{*} \end{array}\right.$$

where *k* is the only gain and, as in any complementary filter, it can be optimally tuned based on the noise characteristics of the signal [26].

So far, direct integration of angular velocities $\omega_{gyr}$ led to a estimation of $R_{IMU}$ complementing the existing estimate $R_{MT}$ from motion capture. Data from accelerometers $a^{T}$, however, cannot be directly integrated because they are expressed in a moving frame and, further more, contain a gravitational bias (In Figure 5, this is expressed by the additional gray-color term $R^{-1}g^{S}$, where *R* is the actual (and unknown) 3D body orientation, estimated by $R_{IMU}$. Because of estimation errors, $R^{-1}R_{IMU}$ is not exactly the identity matrix, this is expressed by the “tilde” variable ${\tilde{g}}^{S}$ which is supposed to be close to $g^{S}$.). By means of the 3D orientation estimates $R_{IMU}$, accelerations can be transformed in space-fixed coordinates, where the gravitational bias can be simply subtracted:

(18) $$a^{S}=R_{IMU}a^{T}-g^{S}$$

as shown in the non-gray area in Figure 5. It should be noted that the gravitational bias is contact in space-fixed frame and equal to $g^{S}={\left[0\;0-9.8\right]}^{T}$ m/s${}^{2}$.

At this point, the space-fixed accelerations (without biased) $a^{S}$ could be directly integrated with respect to time to produce an estimate of linear velocities $v^{S}$. A second integration, in cascade, would lead to an estimation of space-fixed positions $p^{S}$. However, errors in the estimations of the gravitational bias (directly due to attitude errors in $R_{IMU}$) and noise would rapidly lead to a drift of such estimates, in typically rapid time. In order to limit the drift by fusing positions as estimated from the motion capture. The cascade of linear, complementary filter to derive velocity and position estimates is shown in block (iii) of Figure 5.

## 4. Experimental Verification

In this section, the ability of the proposed complementary filter to detect both micro-motions and macro- motions will be demonstrated experimentally. On the one hand, micro-motions entail small amplitude but high-frequency content which is typical of vibratory phenomena. To this end, a 6-degrees of freedom (dof) loadcell will be used as ground-truth to detect accelerations induced by the vibratory phenomena. On the other hand, macro-motions entail large amplitude but low frequency kinematics which is easily detected via motion trackers. We shall show that by fusing both motion-tracking information as well as accelerometers, a complementary filter is able to reliably detect both micro- and macro- motions.

Although the filter structure in Section 2 is general and application-independent, the specific hardware (motion tracker and IMU) along with specific sampling rates, reflects applications involving human motion. In particular, targeting human activities of daily living, the instrumented object was mounted on a leaf-spring and held by one of the authors while performing arbitrary, oscillatory hand-motions. Such motions are deemed representative of activities of daily leaving. The leaf spring mechanism was used to amplify natural and comfortable hand motions, at least in one direction.

**Figure 6:**
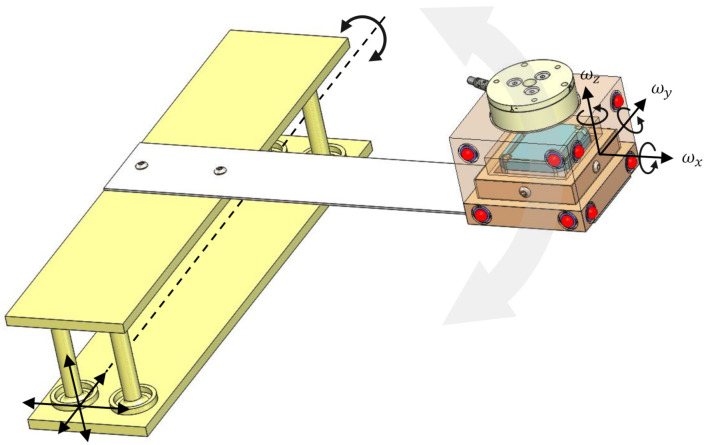
Experimental conditions: Translation and Rotation.

Throughout the experimental condition the IMU, motion capture and loadcell recorded the movement (for more information and animations readers are referred to the supplementary data online). The instrumented object is mounted on a leaf spring allowing only rotations as shown in Figure 6. In this set-up, the instrumented object is attached to a leaf spring fixed to a base. The leaf spring is activated and oscillates until it gets back to its neutral position. Then, the whole setup is moved in space to allow both translations and rotations. In order to validate the complementary filter algorithm, the sampling frequency was set to: 200 Hz, 50 Hz, and 1000 Hz for the IMU, motion capture, and load cell, respectively.

The motion capture acquisition frequency was purposely set to 50 Hz to prove the concept of the complementary filter. The filter algorithm is able to detect the movements which the motion capture cannot such as the vibration with high frequencies and small amplitudes. Motion capture can detect large rotations and translations at lower frequencies but micro-motions may stay undetected when the amplitudes are too small and the frequencies too high.

The measured force of the loadcell is the result of the acceleration and its moments of inertia. They are given by the hardware specifications and a multiplication with the estimated accelerations from the complementary filter will allow us to compare the estimated force with the measured force from the loadcell (see Equation (20)).

Equation (Section 3.2) is time-continuous and any solution starting on SO(3) is guaranteed to remain on SO(3). However, this would no longer be the case if (Section 3.2) were to be directly discretized, e.g., by a numerical simulator, and the solution will likely drift away from SO(3). Campolo et al. [23] proposed a technique that avoids this phenomenon and the following pseudocode in Algorithm 2 describes a possible numerical implementation,
**Algorithm 2:**Complementary filter algorithm on SO(3).
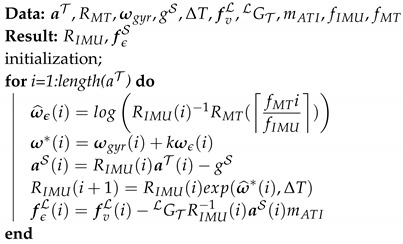

where $f_{IMU}=$ 200 Hz, $f_{MT}=$ 50 Hz is the sampling frequency of the IMU and motion capture, respectively; the notation $\left\lceil \cdot \right\rceil$ denotes the ceiling function maps a real number to the smallest following integer.

The complementary filter uses the motion capture data as reference for the IMU (Figure 5). After each experiment, the estimated acceleration are multiplied with the moments of inertia of the loadcell and compared with the measured forces. The moments inertia of the plate of the ATI mini40 loadcell are

(19) $$m_{ATI}=\left[\begin{array}{ccc} Lxx & Lxy & Lxz\\ Lyx & Lyy & Lyz\\ Lzx & Lzy & Lzz \end{array}\right]=\left[\begin{array}{ccc} 2.99*10^{-2} & -2.27*10^{-4} & 0\\ -2.27*10^{-4} & 3.02*10^{-2} & 0\\ 0 & 0 & 1.79*10^{-2} \end{array}\right]\left[Ns^{2}/m\right]$$

An estimated force from the complementary filter is defined as

(20) $$f_{est}^{L}={}^{L}G_{T}\left(R_{IMU}^{-1}\right)a^{S}m_{ATI}$$

where ${}^{L}G_{T}$ is an homogeneous transformation matrix computed from the CAD of the instrumented box. Therefore, the error between the measured force $f_{v}^{L}$ by loadcell and the estimated force $f_{est}^{L}$ by the complementary filter in the loadcell frame L can be determined as

(21) $$f_{\epsilon }^{L}=f_{v}^{L}-f_{est}^{L}$$

**Figure 7:**
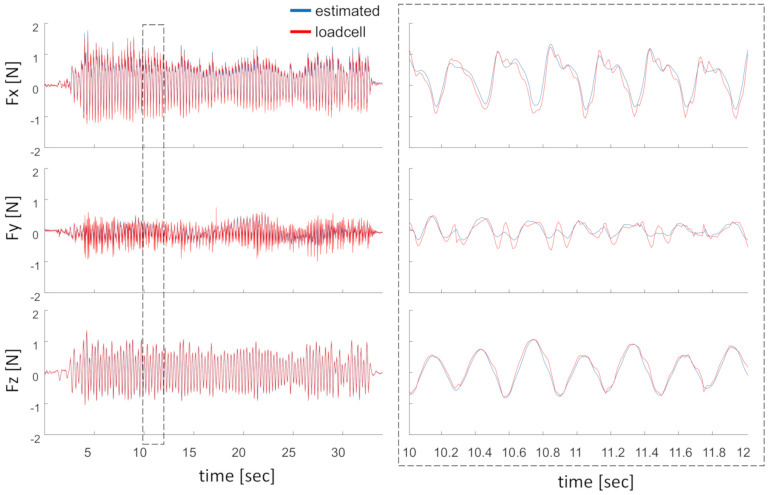
Component-wise comparison of forces as measured from the loadcell and non-inertial forces as estimated through IMU accelerations.

Figure 7 shows how well the estimated forces $f_{est}^{L}$ match the force detected by the loadcell $f_{v}^{L}$. A linear regression of measured forces vs. estimated forces resulted in a goodness of fit with R-squared of 93%, 70%, and 96% for the x-, y-, and z-components, respectively.

**Figure 8:**
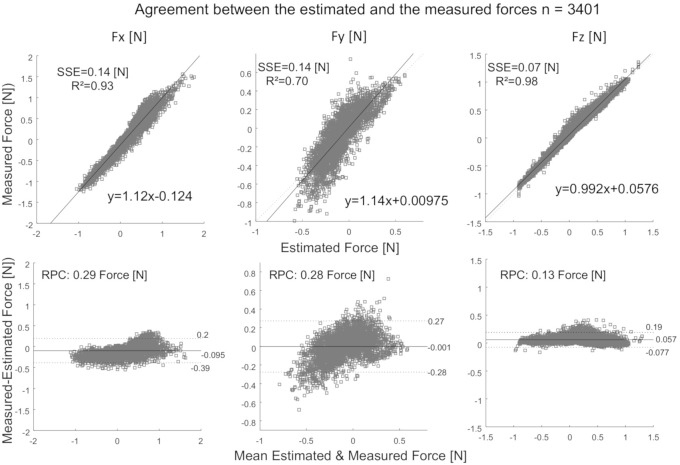
Comparison of the experimental and computed forces (Fx, Fy, Fz) throughout the 34 s experiment (number of data points n = 3401) show strong coherence and small discrepancy between the estimated and measured forces.

Bland–Altman plots [29] were computed to analyze the agreement between the estimated and the measured forces, as shown in Figure 8. Furthermore, the determination coefficient (r2) and the squared sum errors (SSE) were computed for each experiment and each force direction (Fx, Fy, Fz) to measure the discrepancy between the estimated and measured forces. The experimental results show strong coherence and small discrepancy between the estimated and measured forces. The direct comparison between the estimated and measured force shows quasi-linear relationship across all conditions. The complementary filter increases the accuracy of the estimated values of acceleration.

**Figure 9:**
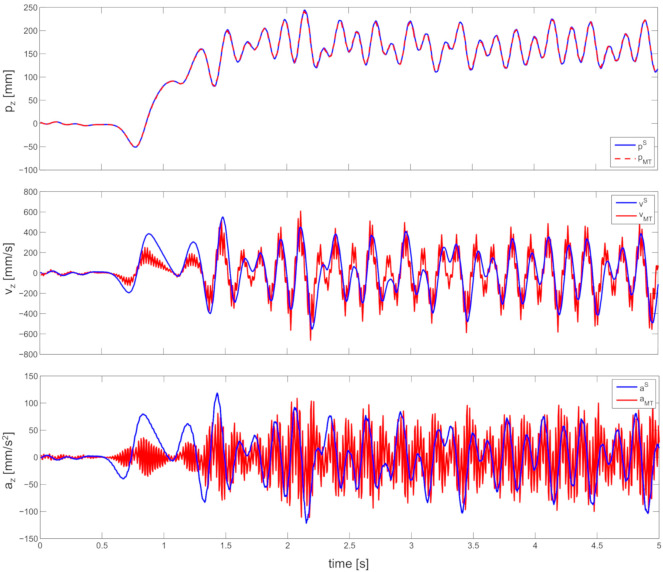
The position and acceleration of the instrumented box in z axis (direction with largest motion).

Finally, Figure 9 shows a comparison of z-axis position, velocity, and accelerations as detected by the motion tracker and as estimated by the SE(3) filter in Figure 5. While position estimates are almost indistinguishable (R-squared >99% when MT positions data are linearly regressed with IMU position estimates), a decay in similarity can be seen when comparing velocities (R-squared <65%) and even more when it comes to accelerations (R-squared <10%).

## 5. Conclusions

In this paper, we have discussed the integration of two measurement systems to detect micro- and macro-motions. Multimodal sensor fusion is conducted using a complementary filter specialized to the nonlinear structure of the SE(3) group of rigid body rotations. This work aimed to improve the estimation of acceleration, free from gravitational bias using a complementary filter. Both the numerical implementation and the experimental validation have been provided as a verification and to complement the theoretical approach. The proposed filter was experimentally tested and validated based on the force measurements from a loadcell.

Optical and IMU technologies can be either used as standalone systems for specific applications but the strength lies in the their combination to deepen our understanding of movement assessment. Optical technology has traditionally been used to the laboratory setting; inertial technology takes movement analysis out to the field. As such, the combination of optical and inertial technologies will contribute to the performance assessments both in sports and industrial tasks.

Potential applications of the proposed approach include clinical movement analysis to identify micro-motions that are clinically relevant [30] as well as tooling task, i.e., capturing kinematics of instrumented tools during manual operations then transferring human skills data to robot [9,31,32].
